# Comparison of Physiologic versus Pharmacologic Mydriasis on Anterior Chamber Angle Measurements Using Spectral Domain Optical Coherence Tomography

**DOI:** 10.1155/2015/845643

**Published:** 2015-03-23

**Authors:** Anna I. Dastiridou, Xiaojing Pan, ZhouYuan Zhang, Kenneth M. Marion, Brian A. Francis, Srinivas R. Sadda, Vikas Chopra

**Affiliations:** ^1^Doheny Image Reading Center, Doheny Eye Institute, Los Angeles, CA 90033, USA; ^2^The Affiliated Hospital of Medical College, Qingdao University, Qingdao, Shandong 266003, China; ^3^Department of Ophthalmology, David Geffen School of Medicine, University of California Los Angeles, Los Angeles, CA 90095, USA

## Abstract

*Purpose*. To compare the effects of physiologic versus pharmacologic pupil dilation on anterior chamber angle (ACA) measurements obtained with spectral domain optical coherence tomography (SD-OCT). *Methods*. Forty eyes from 20 healthy, phakic individuals with open angles underwent anterior segment OCT imaging under 3 pupillary states: (1) pupil constricted under standard room lighting, (2) physiologic mydriasis in a darkened room, and (3) postpharmacologic mydriasis. Inferior angle Schwalbe's line-angle opening distance (SL-AOD) and SL-trabecular-iris-space area (SL-TISA) were computed for each eye and pupillary condition by masked, certified Reading Center graders using customized grading software. *Results*. SL-AOD and SL-TISA under pupillary constriction to room light were 0.87 ± 0.31 mm and 0.33 ± 0.14 mm^2^, respectively; decreased to 0.75 ± 0.29 mm (*P* < 0.01) and 0.29 ± 0.13 mm^2^  (*P* < 0.01), respectively, under physiologic mydriasis; and increased to 0.90 ± 0.38 mm (*P* < 0.01) and 0.34 ± 0.17 mm^2^  (*P* = 0.06) under pharmacologic mydriasis compared to baseline. *Conclusions*. Using SD-OCT imaging, pharmacologic mydriasis yielded the widest angle opening, whereas physiologic mydriasis yielded the most angle narrowing in normal individuals with open iridocorneal angles. Accounting for the state of the pupil and standardizing the lighting condition would appear to be of importance for future studies of the angle.

## 1. Introduction

Anterior segment optical coherence tomography (OCT) has evolved as a new method of imaging the anterior segment. Together with gonioscopy and ultrasound biomicroscopy, it can help in the evaluation and measurement of the anterior chamber angle (ACA) [1, 2]. Early OCT devices, like Visante (Carl Zeiss, Meditec, Dublin, CA, USA), utilized time-domain technology, which provided good visualization of scleral spur but relatively poor localization of Schwalbe's line. The development of spectral domain (SD) OCT provides a faster scan speed and higher resolution imaging of ACA and allows for better visualization of the termination of Descemet's membrane (Schwalbe's line (SL)) and the trabecular meshwork [3–6]. The noncontact nature of OCT can also be advantageous over gonioscopy and ultrasound biomicroscopy in assessing angle anatomy and quantifying the ACA dimensions, since it avoids the inadvertent disruption of the ACA anatomy by the instruments. Additionally, the light levels during the OCT examination can be accurately adjusted, whereas this cannot always be controlled in gonioscopy. As a result, OCT imaging of the anterior segment is a useful tool for angle imaging in clinical practice and can aid in understanding the pathophysiology of angle closure glaucoma [7, 8].

It has long been known that dim light conditions with resultant pupil dilation can be associated with acute attacks of angle closure. Anatomic conditions, such as shallow anterior chamber, shorter axial length, and larger lens volume, are statistically demonstrated risk factors of angle closure glaucoma [9, 10]. However, the anatomic mechanisms taken alone do not explain why the majority of eyes with such predisposing characteristics never develop angle closure glaucoma [11–13]. Recent studies have thus tried to investigate the role of dynamic factors, such as pupil dilation on iris area and iris volume [14–17]. Furthermore, characterizing the effect of lighting variation and pharmacologic mydriasis and cycloplegia on the angle configuration is important, since it could guide in standardizing these conditions for optimal comparison and follow-up of angle anatomy over time. In fact, recent studies have looked at the changes produced by light variation or pharmacologic mydriasis on scleral spur based angle opening. With SD-OCT imaging, the improved visibility of SL allows for better quantification of angle metrics based on that anatomical landmark, instead of the scleral spur [3]. Therefore, because of the ability of SD-OCT to accurately and reproducibly characterize angle anatomy, the goal of our current study was to investigate the effects of pupillary dilation (physiologic versus pharmacologic) on angle measurements.

## 2. Subjects and Methods

Twenty healthy volunteers were recruited for participation in this study. The study was approved by the Institutional Review Board of the University of Southern California, and written informed consent was obtained from all subjects. The research adhered to the tenets set forth in the Declaration of Helsinki.

**Figure 1:**
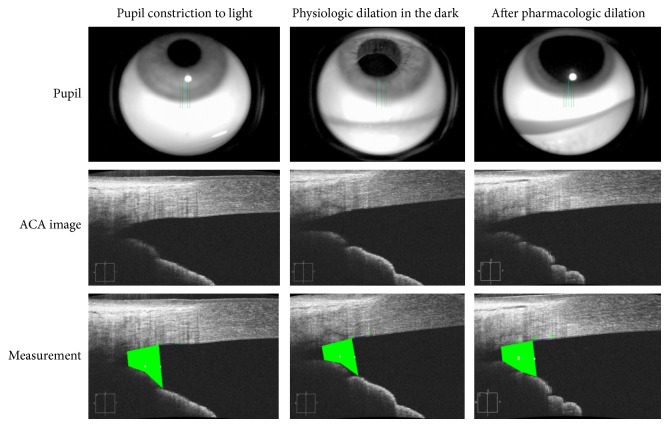
Inferior angle scanning under the three pupillary conditions (ACA: anterior chamber angle).

None of the participants had a systemic or ocular disease history and no one was taking any systemic or topical medications. Age and ethnicity were self-reported. The presence of bilateral normal eyes with open angles was based on ocular examination. Each subject had both eyes (total of 40 eyes) imaged with the Cirrus SD-OCT (software version 6.0.1.24; Carl Zeiss Meditec, Dublin, CA) with the 5-Line Anterior Segment Raster scans protocol, with a scan length of 3 mm. The distance between each of the 5 line scans was 0.25 mm (equaling 1 mm between the 1st and 5th scan). Scans were performed perpendicularly on the inferior angle by centering the 3rd (middle) scan line at the 6 o'clock position of the corneal limbus under three pupillary conditions.  Figure 1 illustrates the inferior angle scanning under the three pupillary conditions. The first set of images was obtained with the pupil constricted under standard room lighting (lights on). The second set of images was obtained on physiologic pupil dilation in a darkened room (lights off with physiologic dilation). The third set of images was obtained thirty minutes after instillation of a drop of 2.5% phenylephrine and 1% tropicamide (pharmacologic dilation). Two sets of images were obtained successively for each pupil condition. The inferior angle SL-angle opening distance (SL-AOD), defined as the distance between SL and the anterior surface of the iris, perpendicular to the corneal endothelium, and the SL-trabecular-iris space area (SL-TISA, measured as the area whose boundaries lie in SL-AOD anteriorly, a line drawn at 500 *μ*m from SL posteriorly, the anterior iris surface, and the trabecular meshwork) were computed for each eye and pupillary condition.

As the Cirrus OCT instrument does not yet include angle measurement tools, images were exported from the instrument and analyzed using the National Institutes of Health image-analysis software (Image J 1.44p; developed by Wayne Rasbands, National Institutes of Health, USA). All grading and computation of angle metrics were performed by two independent, certified anterior segment OCT graders (XP, JZ) at the Doheny Image Reading Center.

The mean of two successive acquisitions was used for each pupillary condition and differences in SL-AOD and SL-TISA between different pupillary conditions were evaluated by repeated measures ANOVA analysis, accounting for inclusion of both eyes from each participant in the calculations. Post hoc analysis was performed for pairwise comparisons between measurements in the light, in the dark, and after pharmacologic pupil dilatation. Correlation analysis was performed with computation of Pearson's correlation coefficients. Significance was set at *P* < 0.05. All statistical analyses were performed using Statistical Package for Social Science (version 18.0; SPSS Inc., Armonk, NY).

## 3. Results

Eleven female and 9 male participants were enrolled. The mean age was 32.3 ± 7.4 (24 to 53) years and the majority of participants were of Asian descent (65%).

All eyes were imaged in the three different conditions. The mean values for the inferior angle metrics from the different pupillary conditions (pupil constricted under standard room lighting, physiologic mydriasis in a darkened room, and after pharmacologic mydriasis) are shown in Table 1. Pupil diameter was 4.00 ± 0.81 mm increasing to 5.27 ± 0.77 mm (*P* < 0.001) in the darkened room examinations and to 6.54 ± 0.76 mm (*P* < 0.001) after pharmacologic dilation. SL-AOD under pupillary constriction to room light was 0.87 ± 0.31 mm, decreasing by 0.13 ± 0.12 mm under physiologic mydriasis (*P* < 0.001) and increasing by 0.09 ± 0.20 mm (*P* = 0.001) under pharmacologic mydriasis. Analogously, SL-TISA under pupillary constriction to room light measured 0.33 ± 0.14 mm^2^, decreasing by 0.05 ± 0.05 mm^2^ (*P* < 0.001) under physiologic mydriasis, whereas the change between the measurements under pharmacologic mydriasis compared to the baseline did not reach statistical significance (0.03 ± 0.08 mm^2^, *P* = 0.057).

Finally, we assessed whether there was an association between the magnitude of SL-AOD changes in relation to the SL-AOD tested with the room lights on. There was no correlation between the amount of the change in SL-AOD from light to dark (*P* = 0.093) or the change in SL-AOD from light to dilation (*P* = 0.685) and the value of SL-AOD in the room lights testing. However, there was a statistically significant correlation between the change in SL-AOD and the change in pupil diameter from light to dark (*P* = 0.010). This relation was not significant between the change in SL-AOD and the respective pupil diameter change comparing between the room lights examination and that after pharmacologic dilation (*P* = 0.663).

## 4. Discussion

In the present study, we evaluated the dynamic changes of the inferior ACA metrics in phakic healthy volunteers using the Cirrus SD-OCT under three pupillary conditions. Our results support an angle opening effect with bright illumination, as well as with pharmacologic mydriasis, and angle narrowing with dim illumination-associated physiologic dilation. Additionally, our study provides evidence that lightning conditions should be considered when angle imaging is performed, especially with anterior segment OCT, where the illumination conditions can more accurately and more easily be controlled, compared to other angle assessment modalities like gonioscopy. Accounting for the state of the pupil and standardizing the lighting condition would appear to be of importance for future studies of the angle.

Importantly, in this study, we have documented the changes that occur in SL-based angle metrics imaged with SD-OCT. Since the SL is much more easily identified than scleral spur (SS) with the SD-OCT systems, SL-based AOD and TISA are often preferred to the SS-based AOD500 or TISA500 with these newer OCT devices [3, 18]. However, the existing data in the literature on angle configuration changes with light and dilation come mainly from studies reporting on scleral spur based metrics (AOD500 and AOD750) with the use of the time-domain Visante OCT [15, 19–23] and the swept source OCT Casia [23]. In fact, the AOD500 and 750 metrics were originally introduced because the scleral spur was easily identifiable in UBM and anterior segment OCT systems operating at longer wavelengths, based on an approximate distance between the scleral spur and SL. Overall, SL-AOD might represent a more meaningful metric in angle studies compared to AOD500 [3, 18].

Previous studies have investigated the effect of lighting variation and physiologic dilation on the angle opening with variable findings. Leung et al. were the first to evaluate the dark-light changes on the nasal angle with anterior segment OCT, showing a linear change in AOD with change in pupil diameter in almost all of the eyes studied [19]. Wang et al. quantified the effect of lighting conditions on AOD500 and TISA500 in different ethnic groups and demonstrated a greater dark to light change in both parameters in ethnic Chinese compared to Caucasians [24]. In a study by Aptel et al. [20], the AOD500 measured with the time-domain Visante OCT decreased by 20% in open-angle eyes when comparing measurements in bright light to those after physiologic dilation in the dark. In that study, interestingly, the authors found the iris volume change per millimeter of pupil dilation to correlate significantly with AOD500 decrease after pupil dilation. In another recent study, Masoodi et al. used the Spectralis SD-OCT and reported a significant decrease in the nasal and temporal AOD500 when comparing AOD500 under light and dark conditions [25].

Furthermore, only a few studies have also quantified the effect of pharmacologic dilation, in addition to that of lighting variations, on ACA metrics. In a study by Aptel and Denis [15] the authors evaluated differences in angle and iris parameters and reported similar AOD500 and TISA500 measures under bright lighting conditions, after pharmacologic mydriasis with phenylephrine and after tropicamide instillation in open-angle eyes. Iris area and volume decreased significantly after either phenylephrine or tropicamide drops. It is interesting that, despite the increase in pupil diameter, the angle opening which measured 500 *μ*m from the scleral spur did not change. However, a statistical significant decrease in AOD and TISA, along with an increase in iris volume, was found for the fellow eyes of narrow-angle eyes when comparing images under bright light conditions to those after phenylephrine or tropicamide instillation. In a large cohort in Chinese patients, where imaging was performed under light, under dark, and after pharmacologic dilation, again with the Visante OCT, although the authors do not report the statistical analysis for AOD and TISA comparisons, interestingly there is virtually no difference in AOD500 from light to dark, but there is an increase in AOD500 after pharmacologic mydriasis [21]. In a recent study with a swept source OCT, the open-angle glaucoma and control groups developed little or virtually no change in AOD750 between the bright room and dark room imaging, whereas, after dilation, the AOD750 increased significantly [26]. Finally, in a study in cataract patients, again with the OCT Casia, the authors reported variable changes in angle opening after pharmacologic mydriasis [27]. Due to differences in instruments and computed angle metrics, there cannot be a direct comparison between the above studies and the present one. Rather, our study suggests that SL-AOD decreases when the pupil dilates in a darkened room and it increases after pharmacologic mydriasis, compared to physiologic pupil constriction under standard room lighting.

Cross-sectional studies have also investigated changes in iris area and iris volume with changes in light conditions and after dilation, in order to evaluate the dynamic changes in the anterior segment and how they could contribute to the pathogenesis of primary angle closure [14, 28, 29]. Quigley et al., using AS-OCT, found that the iris cross-sectional area is nearly two times smaller after physiologic or pharmacologic pupil dilation in healthy eyes and that a lower reduction of the iris cross-sectional area after pupil dilation may be a potential risk factor for angle closure [14]. The authors hypothesized that the normal iris loses volume in the dark or after pharmacologic pupil dilation and that eyes with angle closure lose less iris volume on dilation, contributing to iridotrabecular apposition. In addition, Aptel and Denis demonstrated that iris volume increases after pupil dilation in narrow-angle eyes predisposed to acute angle closure, whereas it decreases in open-angle eyes [15]. Longitudinal studies evaluating whether iris volume change with darkness could be used to identify narrow-angle eyes that will finally develop angle closure would be required to validate this hypothesis. Also, it would be interesting to prospectively evaluate whether peripheral iris configuration and angle opening changes when going from light to dark could be used to identify narrow-angle eyes that need a prophylactic iridotomy. Consequently, based on published studies, in eyes with open angles on gonioscopy, when the pupil increases, under both physiologic and pharmacologic dilation, the iris area and volume decrease [14, 15, 19, 21]. Additionally, based on our results, the distance from SL to the iris decreases in the dark, whereas in mydriasis, the SL-AOD increases. This is important in the effort to characterize the change in anteroposterior dimensions and angle opening as the iris muscles contract and dilate.

Since in the present study we quantified the magnitude of the changes in angle opening in open-angle eyes, it remains unknown whether SL-AOD decreases or increases with differences in light and after pharmacologic dilation in narrow-angle patients. Studies have looked at changes in AOD500 between narrow angles and controls and showed that it decreases both from light to dark [19, 20] and from light to pharmacologic mydriasis [15]. This may indicate a differential response for the narrow angle for SL-AOD also. It is important that, in a narrow-angle eye, the angle closure attacks are precipitated by pupil dilation due to dim illumination, particularly in the mid-dilated position at which there is maximum resistance to aqueous flow between the iris and the lens. Since anatomical parameters, such as anterior chamber depth, axial length, or lens position and thickness, alone, do not differentiate the eyes that will eventually develop angle closure, it was suggested that it is the physiological response of some eyes in addition to their anatomy that predisposes them to angle closure [6].

The narrowing of the angle under dark conditions could be attributed to the relative changes in the dimensions of the iris-lens channel, as well as the changes in peripheral iris configuration, which occur with pupil dilation [30]. It has also been suggested that the volume of the iris changes by eliminating extracellular fluid, based on the high fluid content of iris stroma and the ability of water to pass through it [14]. On the other hand, the angle opening effect inspected in normal volunteers after instillation of phenylephrine and tropicamide drops could be explained by the resultant cycloplegia, which produces a posterior displacement of the iris-lens diaphragm. Other parameters that are supposed to account for the changes in iris configuration could be the dilating force that pulls the iris towards the trabeculum and tends to produce a more convex peripheral iris configuration and the vasoconstrictive action of phenylephrine on iris vessels. It is therefore interesting to note that Aptel and Denis found no differences between the effects of phenylephrine and tropicamide on AOD 500 or TISA500, which would suggest a change in the iris bowing or thickness [15].

Investigating quantitative change in angle configuration after pupil dilation depends on precise recording of the angle width. Cirrus SD-OCT has been shown to provide repeatable measurements of the angle and as documented in previous studies from our group, the intergrader reproducibility was also excellent, allowing us to measure the ACA quantitatively [30]. Moreover, while for each set of scans, the 6 o'clock position scan was chosen for analysis, a previous study from our group suggested that small deviations in the angle of the scan do not introduce significant errors in the SL-AOD and SL-TISA measures [31]. In addition, the same measurement series was followed for all patients, first under room lights, then under dark conditions, and finally 30 minutes after pharmacologic dilation. It has indeed been suggested that the change in iris configuration occurs very rapidly and that the iris area assumes a stable value in seconds after moving in the dark [14]. As a result, we did not randomize the series of testing conditions between light and dark. Also, as the main objective of our study was to describe the dark-light changes in ACA metrics, we sampled only the inferior angle, with the assumption that the dynamic change in ACA metrics is similar in other quadrants. A recent study supports this assumption, at least in open-angle eyes, where there appears to be no significant variation in AOD500 in the different quadrants in the dark [23].

## 5. Conclusions

Inferior angle ACA metrics decrease with physiologic pupil dilation and increase after pharmacologic pupil dilation in normal eyes, indicating that the iris is a dynamic structure, constantly changing in configuration in response to light and drug stimuli. ACA metrics differed significantly based on the lighting condition and the state of pupillary dilation. Standardizing of lighting conditions should be considered for objective measurement of ACA metrics in clinical trials and clinical practice. In addition, investigating angle width and iris dynamic changes with light condition could provide important information in understanding the mechanism of primary angle closure. Further studies are warranted to study the dynamic response of the narrow-angle eye to light and drug stimuli and whether this change in SL-AOD could constitute an objective parameter to assess the risk for iris trabecular apposition.

## Figures and Tables

**Table 1:** Effect of differing lighting conditions and pharmacologic dilation on pupil size and angle metrics obtained with Cirrus. Results are presented as mean ± standard deviation.

	Light	Dark	Pharmacologic dilation	*P* value
Pupil size (mm)	4.00 ± 0.81	5.27 ± 0.77	6.54 ± 0.76	overall: <0.001^1^ (1)-(2): <0.001^2^ (1)–(3): <0.001^2^ (2)-(3): <0.001^2^

SL-AOD (mm)	0.87 ± 0.31	0.75 ± 0.29	0.9 ± 0.38	overall: <0.001^1^ (1)-(2): <0.001^2^ (1)–(3): 0.001^2^ (2)-(3): <0.001^2^

SL-TISA (mm^2^)	0.33 ± 0.14	0.29 ± 0.13	0.34 ± 0.17	overall: 0.003^1^ (1)-(2): <0.001^2^ (1)–(3): 0.057^2^ (2)-(3): <0.001^2^

^1^Repeated measures, ANOVA.

^2^Post hoc pairwise comparisons.

SL-AOD: Schwalbe's line-angle opening distance.

SL-TISA: Schwalbe's line-trabecular-iris space area.
